# β_3_ adrenergic receptor in the kidney may be a new player in sympathetic regulation of renal function

**DOI:** 10.1016/j.kint.2016.03.020

**Published:** 2016-09

**Authors:** Giuseppe Procino, Monica Carmosino, Serena Milano, Massimo Dal Monte, Giorgia Schena, Maria Mastrodonato, Andrea Gerbino, Paola Bagnoli, Maria Svelto

**Affiliations:** 1 Department of Biosciences, Biotechnologies and Biopharmaceutics, University of Bari, Bari, Italy; 2 Department of Sciences, University of Basilicata, Potenza, Italy; 3 Department of Biology, University of Pisa, Pisa, Italy; 4 Department of Biology, University of Bari, Bari, Italy; 5 Institute of Biomembranes and Bioenergetics, National Research Council, Bari, Italy; 6 National Institute of Biostructures and Biosystems (INBB), Rome, Italy

**Keywords:** antidiuresis, AQP2, β_3_ adrenergic receptors, NKCC2, vasopressin

## Abstract

To date, the study of the sympathetic regulation of renal function has been restricted to the important contribution of β_1_- and β_2_-adrenergic receptors (ARs). Here we investigate the expression and the possible physiologic role of β_3_-adrenergic receptor (β_3_-AR) in mouse kidney. The β_3_-AR is expressed in most of the nephron segments that also express the type 2 vasopressin receptor (AVPR2), including the thick ascending limb and the cortical and outer medullary collecting duct. *Ex vivo* experiments in mouse kidney tubules showed that β_3_-AR stimulation with the selective agonist BRL37344 increased intracellular cAMP levels and promoted 2 key processes in the urine concentrating mechanism. These are accumulation of the water channel aquaporin 2 at the apical plasma membrane in the collecting duct and activation of the Na-K-2Cl symporter in the thick ascending limb. Both effects were prevented by the β_3_-AR antagonist L748,337 or by the protein kinase A inhibitor H89. Interestingly, genetic inactivation of β3-AR in mice was associated with significantly increased urine excretion of water, sodium, potassium, and chloride. Stimulation of β3-AR significantly reduced urine excretion of water and the same electrolytes. Moreover, BRL37344 promoted a potent antidiuretic effect in AVPR2-null mice. Thus, our findings are of potential physiologic importance as they uncover the antidiuretic effect of β_3_-AR stimulation in the kidney. Hence, β_3_-AR agonism might be useful to bypass AVPR2-inactivating mutations.

In the kidney, the antidiuretic hormone arginine vasopressin (AVP) is a critical regulator of water and electrolyte homeostasis. AVP is released from the pituitary gland into the bloodstream and binds to the type 2 vasopressin receptor (AVPR2),^1^ a G protein–coupled receptor localized in the thick ascending limb of Henle, the distal convolute tubule, and the collecting duct, acting mainly through the cAMP–protein kinase A pathway.

In the thick ascending limb of Henle, AVP stimulates NaCl reabsorption across the Na-K-Cl cotransporter (NKCC2), increasing its phosphorylation,^2^ thus generating the corticomedullary osmotic gradient providing the driving force for water reabsorption in the kidney tubules.

In the CD, AVP stimulates the exocytosis of the water channel aquaporin 2 (AQP2)^3^ at the apical membrane of the principal cells, dramatically increasing water reabsorption (for a review, see ref. 4). Inactivating mutations of the *AVPR2* gene cause X-linked nephrogenic diabetes insipidus (XNDI), characterized by constant diuresis and the risk of severe dehydration.^5^ Many studies have shown that hormones other than AVP also exhibit antidiuretic effect,^6^, ^7^, ^8^, ^9^, ^10^ suggesting novel strategies to manage XNDI.

The β-adrenergic system controls several renal functions. In particular, types 1 and 2 β-adrenoreceptors (β_1-2_-AR)^11^ regulate renal blood flow, glomerular filtration rate (GFR), sodium and water reabsorption, acid-base balance, and secretion of renin (for a review, see Johns *et al.*^12^).

Among β-ARs, the β_3_-AR is the last identified member of this family. At first, it was shown to regulate lipolysis and thermogenesis in adipose tissue,^13^ whereas subsequently it was shown to play important roles in the pathophysiology of the cardiovascular^14^ and urinary^15^ systems. However, its expression and possible physiologic role in the kidney remains to be fully clarified. There are indications in mice that β_3_-AR mRNA is expressed by renal arteries.^16^ In addition, in the rat kidney, a cDNA microarray screening showed that β_3_-AR is expressed in the kidney medulla.^17^ Moreover, in humans, β_3_-AR polymorphisms seem to be associated with the effect of thiazide diuretics,^16^, ^18^ suggesting a role for β_3_-AR in regulating renal water reabsorption. In this respect, demonstrating this novel role of β_3_-AR in renal physiology is particularly intriguing in light of potential therapeutic applications of β_3_-AR–acting drugs in diseases characterized by altered diuresis. Moreover, β_3_-AR is relatively resistant to agonist-induced desensitization,^19^ which would ensure prolonged pharmacologic stimulation *in vivo*. In addition, due to the limited number of tissues expressing β_3_-AR, compared with β_1-2_-AR, β_3_-AR agonists are supposed to show a low systemic off-target effect.^14^

## Results

### β_3_-AR expression in the mouse kidney

**Figure 1:**
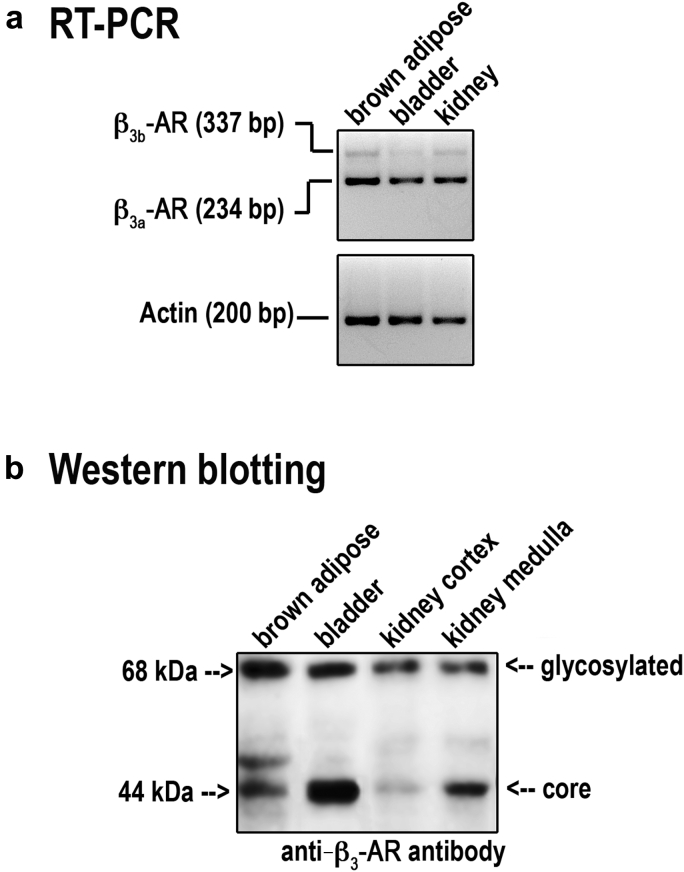
**Expression of β**_**3**_**-ARs mRNA and protein in mouse kidney.** (**a**) Total RNA from mouse kidney was probed for the presence of mRNA coding β_3_-adrenergic receptors (β_3_-ARs). Brown adipose tissue and bladder were used as positive controls. Two amplicons corresponding to β_3b_-AR (337 bp) and β_3a_-AR (234 bp) were visualized in all samples. Control reverse transcriptase polymerase chain reaction was performed using primers amplifying mouse β-actin. (**b**) Total protein extracts from mouse kidney cortex and total medulla were analyzed by Western blotting using anti–β_3_-AR antibodies. Two bands, corresponding to the core and the glycosylated protein, were detected in the kidney fractions at the same molecular size as those revealed in brown adipose and urinary bladder. Experiments were repeated 3 times with comparable results. RT-PCR, reverse transcriptase polymerase chain reaction.

Reverse transcriptase polymerase chain reaction revealed that β_3_-AR mRNA was clearly detectable in the RNA samples from the mouse kidney, brown adipose tissue, and bladder (Figure 1a). In particular, the intron-spanning primers amplified 2 bands of 234 bp and 337 bp, representing β_3a_-AR and β_3b_-AR transcripts, respectively.^20^ Sequencing confirmed the specificity of the obtained bands (data not shown).

Immunoblotting analysis revealed that mouse kidney cortex and total medulla expressed a band of 44 kDa for the core protein and 1 at 68 kDa for the glycosylated form in all samples (Figure 1b). Both bands were also revealed in β_3_-AR–expressing control tissues.

### Immunolocalization of β_3_-ARs in the mouse kidney

**Figure S1:**
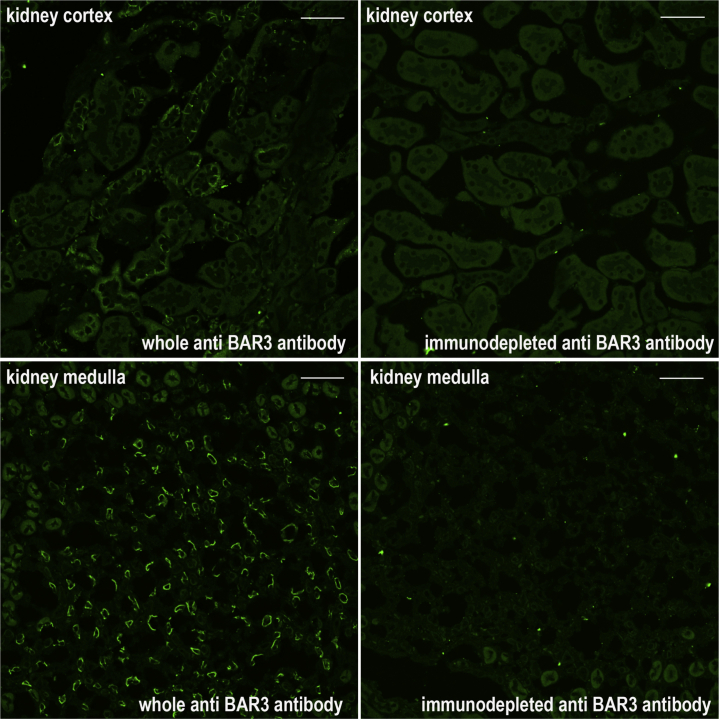
Pre-adsorption of anti β3-AR antibody on its immunizing peptide completely abolished the immunostaining of β3-AR in mouse kidney sections. Goat antiβ3-AR (#SC1473) was preadsorbed on its immunizing peptide (SC1473p) and used to immunostained paraffin-embedded mouse kidney sections. Compared to whole antibody, immunodepleted antibody failed to detect β3-AR-positive tubule in both kidney cortex and medulla (bar = 50 μm).

**Figure 2:**
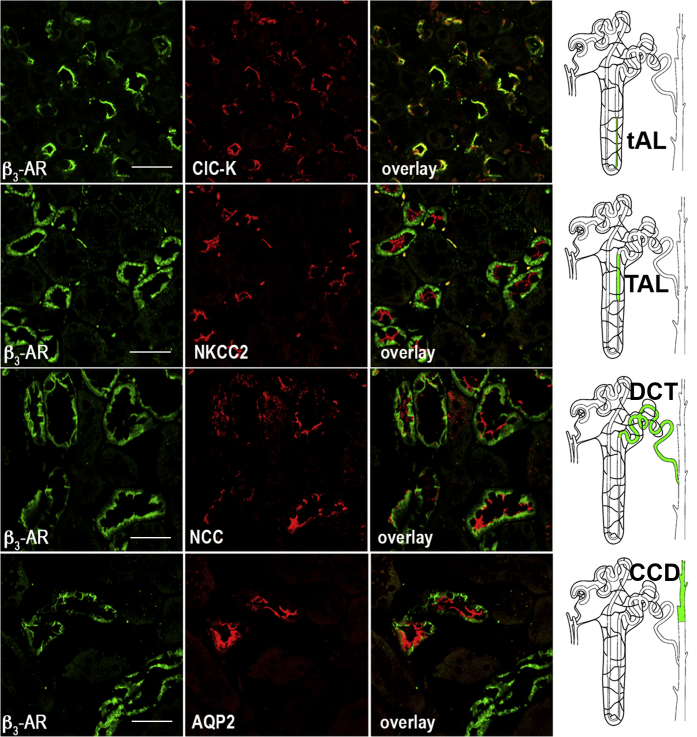
**Immunolocalization of β**_**3**_**-AR in mouse kidney.** Paraffin-embedded kidney sections (C57BL6/J, wild type) were immunostained with anti–β_3_-adrenergic receptor (β_3_-AR) antibodies (green) and costained with antibodies against specific markers of different segments of the kidney tubule: kidney-specific chloride channel (CLC-K) channel for the thin ascending limb (tAL), Na-K-Cl cotransporter (NKCC2) for the thick ascending limb (TAL), NaCl symporter (NCC) for the distal convolute tubule (DCT), and aquaporin 2 (AQP2) for the cortical collecting duct (CCD) (all in red). Overlay of the each double-staining experiment indicated significant expression of β_3_-AR in the tAL, TAL, DCT, and CCD. Drawings of the nephron on the right show in light green the β_3_-AR–positive segments. The same results were obtained in 5 different animals (bar = 20 μm).

As shown in Figure 2, β_3_-AR was expressed at the apical and basolateral membrane of the epithelial cells of the thin ascending limb, identified by the presence of the kidney-specific chloride channel ClC-K1.^21^, ^22^ β_3_-AR was also localized at the basolateral membrane of the epithelial cells of (i) the thick ascending limb of Henle, expressing the apical NKCC2 cotransporter^23^; (ii) the distal convolute tubule, expressing the apical thiazide-sensitive NaCl symporter (NCC)^24^; and (iii) the cortical CD and the outer medullary CD (the latter not shown), expressing AQP2 at the apical membrane.^25^ The staining for β_3_-AR completely disappeared when the anti–β_3_-AR antibody used for immunofluorescence was preadsorbed on its immunizing peptide (Supplementary Figure S1).

**Figure S2:**
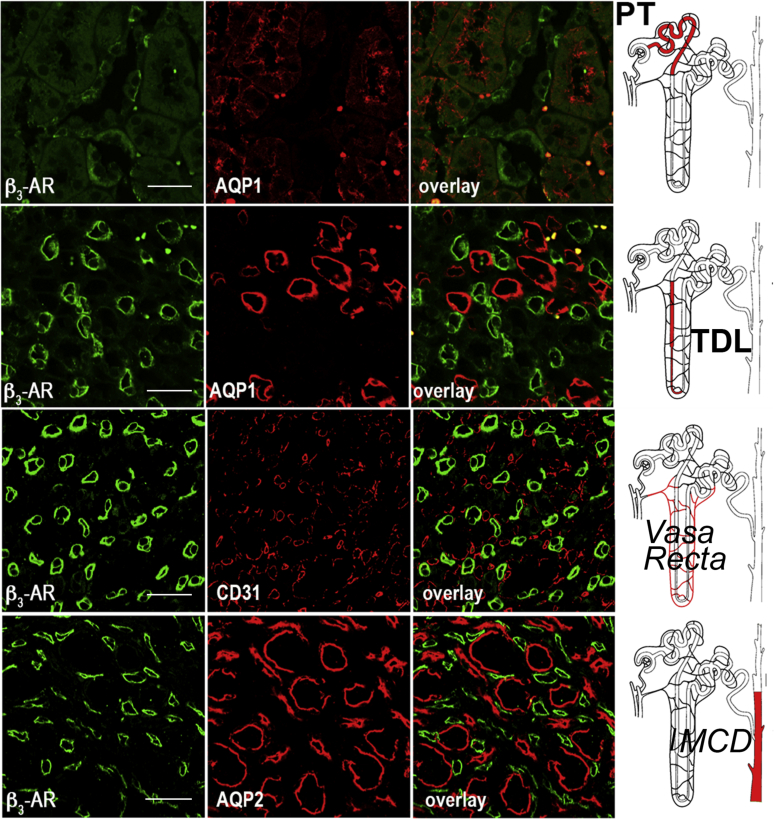
Immunolocalization of β3-AR in mouse kidney. Paraffin-embedded kidney sections (C57BL6/J, wt) were immunostained with anti β3-AR antibodies (green) and co-stained with antibodies against specific markers of different segments of the kidney tubule or vasculature: AQP1 for the proximal tubule (PT) and the thin descending limb (TDL), AQP2 for the inner medullary collecting duct (IMCD) and CD31 for the endothelium of *Vasa Recta* (all in red). Overlay of the each double staining experiment indicated that β3-AR was neither expressed in the PT nor in the TDL nor in the IMCD nor in the *Vasa Recta*. Drawings of the nephron on the right column indicated in red the β3-AR-negative tubule or vascular portions. β3-AR was expressed at the basolateral plasma membrane in all the tubules where it is expressed. Same results were obtained in at least 5 animals (bar = 20 μm).

We also demonstrated that β_3_-AR was neither expressed in the proximal convolute tubule nor in the thin descending limb of Henle’s loop, the inner medullary CD, and the *vasa recta* (Supplementary Figure S2). Overall, the current data show that β_3_-AR is localized in those nephron tracts also expressing AVPR2.

### Effect of β_3_-AR activation on cAMP production, AQP2 trafficking, and NKCC2 phosphorylation: *ex vivo* experiments

**Figure 3:**
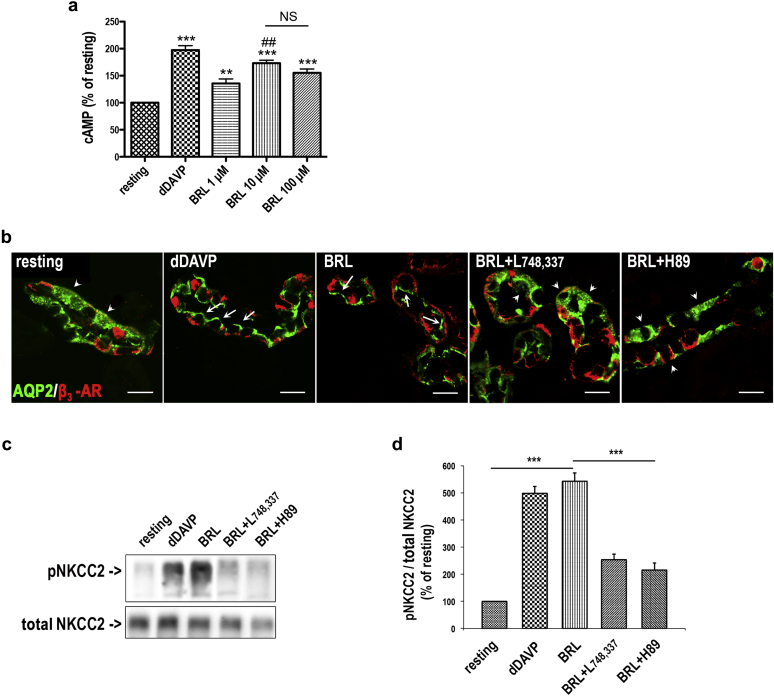
***Ex vivo* β**_**3**_**-AR activation in a kidney tubule: intracellular cAMP measurements, AQP2 subcellular localization, and NKCC2 phosphorylation.** (**a**) 1-Deamino-8-d-arginine-vasopressin (dDAVP)– and BRL37344 (BRL)-induced cAMP production in mouse kidney tubule suspensions. Freshly isolated tubule suspensions from wild-type mice (12-week-old males, 3 per individual experiment) were pooled and equally distributed into 24-well plates. Samples were treated with dDAVP (10^−7^ M) or with the indicated concentrations of BRL for 60 minutes at 37 °C. Total cAMP-generated in each well was normalized to the protein content. Three independent experiments were carried out. Data are expressed as the percentage of the cAMP content measured in resting cells ± SEM. Significant differences between means were tested by 1-way analysis of variance with the Newman-Keuls posttest. Significance was accepted for *P* values < 0.05. ***P < 0.0001, **P < 0.001 compared with resting tubules. ##*P* < 0.001 compared with 1 μM BRL. (**b**) Freshly isolated kidney slices (250 μm) were rapidly cut after sacrifice, maintained in CO_2_-equilibrated culture medium at 37 °C, left untreated (resting), or incubated with dDAVP (10^−7^ M) or with BRL (10 μM BRL). BRL was also incubated after preincubation with either the β_3_-AR–antagonist (L748,337, 10^−7^ M) or the protein kinase A inhibitor (H89) (10^−5^ M). Slices were treated as described and fixed, and ultrathin sections were stained for aquaporin 2 (AQP2) and β_3_-adrenergic receptor (β_3_-AR) and subjected to confocal microscopy. BRL was as effective as dDAVP in promoting AQP2 expression at the apical plasma membrane of cortical and outer medullary collecting duct cells (white arrows) compared with the intracellular localization of AQP2 observed in untreated samples (resting) or samples incubated with BRL after preincubation with L748,337 or H89 (white arrowheads) (bar = 15 μm). (**c**) Kidney slices were treated as described, then lysed, and total protein extracts underwent Western blotting analysis using the anti–phosphorylated Na-K-Cl cotransporter (pNKCC2) and the anti–total NKCC2 antibodies. (**d**) Densitometric analysis showed a five-fold increase in pNKCC2 (normalized to total NKCC2) in samples treated with BRL or dDAVP, and the effect of BRL was significantly prevented by L748,337 and H89. Data are provided as mean ± SEM and expressed as a percentage of the resting condition. Significant differences between means were tested by 1-way analysis of variance with the Newman-Keuls posttest. ***P < 0.001. Comparable results were obtained in 3 different mice. NKCC2, Na-K-Cl cotransporter; NS, not significant.

Our finding that β_3_-AR is expressed in the AVPR2-positive kidney segments prompted us to investigate whether β_3_-AR activation may mimic the effect of AVP on cAMP production, AQP2 intracellular trafficking, and NKCC2 activation. Using an *ex vivo* model consisting of freshly isolated mouse kidney tubule suspensions, we measured changes in intracellular cAMP concentrations in response to either the specific β_3_-AR agonist BRL37344 (1, 10, 100 μM) or the AVP analog 1-deamino-8-d-arginine-vasopressin (dDAVP), 10^−7^ M, used as positive control for cAMP production (Figure 3a). Results are reported as the percentage of the cAMP concentration measured in resting tubules. Treatment with BRL37344 led to a concentration-dependent increase in intracellular cAMP levels, with the maximal effect observed at 10 μM (+173%, *P* < 0.0001).

Accordingly, we used 10 μM BRL37344 for all the following experiments performed in freshly isolated live mouse kidney slices, untreated (resting) or incubated with either dDAVP or BRL37344 (Figure 3b). Confocal microscopy showed that both BRL37344 and dDAVP promoted AQP2 accumulation at the luminal plasma membrane of cortical collecting duct cells (Figure 3b, white arrows) compared with the cytoplasmic localization of AQP2 observed in control slices (Figure 3b, white arrowheads). In line with the absence of β_3_-AR in the inner medullary CD, BRL37344 failed to induce AQP2 apical accumulation in this portion of the CD (not shown). Importantly, the effect of BRL37344 was prevented by preincubation with either the β_3_-AR-selective antagonist L748,337^26^ or the protein kinase A inhibitor H89.^27^

Next, we evaluated the level of NKCC2 phosphorylation under the same experimental conditions using an antibody against the regulatory phosphothreonine residues in the N-terminus of NKCC2.^28^ Western blotting (Figure 3c and d) showed that p-NKCC2 increased by about 5-fold after BRL37344 treatment compared with resting conditions, an effect comparable to that obtained with dDAVP. Pretreatment with either L748,337 or H89 significantly prevented this effect of BRL37344. Of note, incubation of kidney slices with either L748,337 or H89 alone did not change AQP2 subcellular localization or NKCC2 phosphorylation compared with resting slices (not shown).

**Figure 4:**
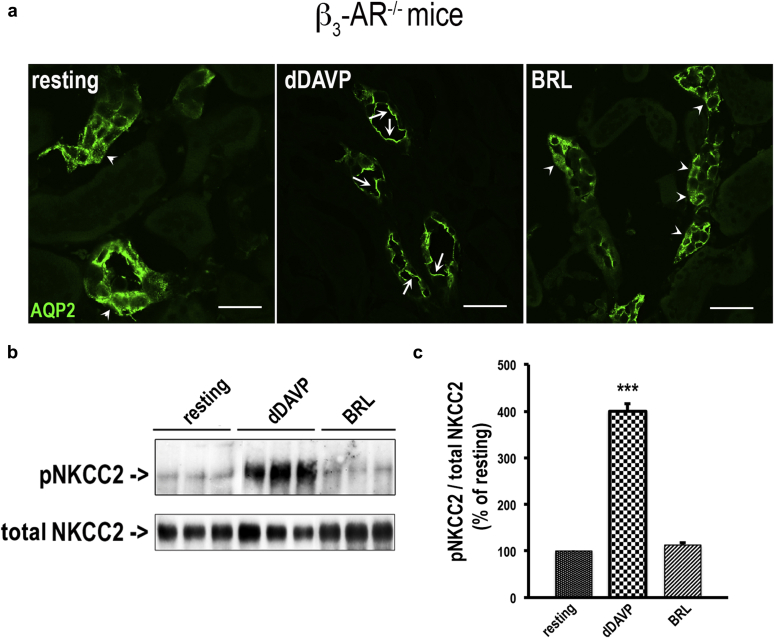
**BRL37344 failed to induce AQP2 apical expression and NKCC2 phosphorylation in the kidney of β**_**3**_**-AR–null mice.** (**a**) Freshly isolated kidney slices (250 μm) were obtained from β_3_^−/−^AR (β_3_-adrenergic receptor) mice, maintained in CO_2_-equilibrated culture medium at 37 °C, and left untreated (resting) or incubated with desmopressin (dDAVP, 10^−7^ M) or BRL37344 (BRL, 10 μM). Slices were fixed, and ultrathin sections (5 μm) were stained for AQP2 (aquaporin 2) and subjected to confocal laser-scanning microscopy. In β_3_^−/−^AR mice, BRL was unable to promote AQP2 expression at the apical plasma membrane of cortical and outer medullary collecting duct cells. dDAVP was used as an internal control to promote AQP2 apical expression (bar = 10 μm). Arrows indicate apical plasma membrane staining. Arrowheads indicate intracellular staining. (**b**) Slices were also lysed and protein extracts subjected to Western blotting analysis with antiphosphorylated Na-K-Cl cotransporter (pNKCC2) and total Na-K-Cl cotransporter (NKCC2) antibodies. (**c**) Densitometric analysis of pNKCC2, normalized to total NKCC2, showed that in β_3_^−/−^AR mice, BRL was unable to increase NKCC2 phosphorylation compared with dDAVP. Data are provided as mean ± SEM and expressed as the percentage of the resting condition. Significant differences between means were tested by 1-way analysis of variance with the Newman-Keuls posttest. ***P < 0.0001. Comparable results were obtained in 3 different mice.

To confirm that these effects of BRL37344 were ascribable to β_3_-AR stimulation, we repeated these experiments on live kidney slices from β_3_-AR–null mice (β_3_-AR^−/−^).^29^ Importantly, in the absence of β_3_-AR functional expression, BRL37344 promoted neither AQP2 apical accumulation (Figure 4a) nor NKCC2 phosphorylation (Figure 4b and c). In addition, in β_3_-AR^−/−^ mice, 10 μM BRL37344 was unable to promote intracellular cAMP elevation in isolated kidney tubules (not shown).

### Effect of β_3_-AR knockout on water and electrolyte handling in the mouse kidney

**Figure 5:**
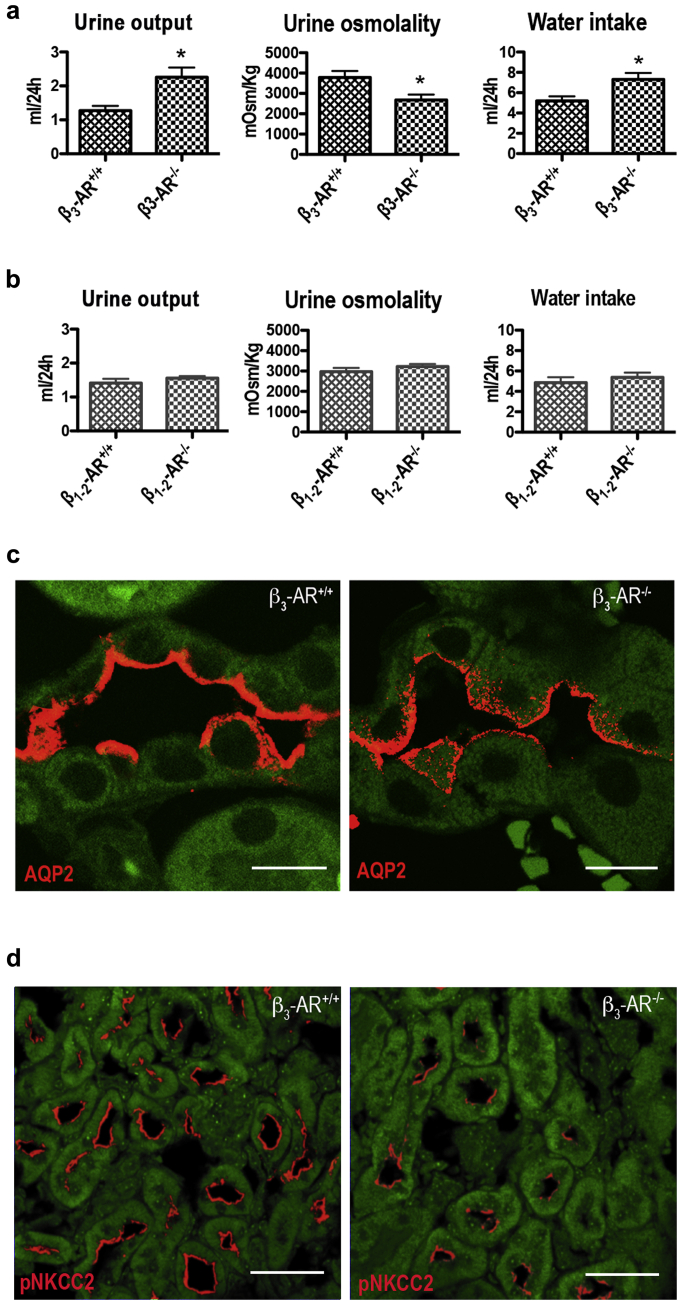
**Mice lacking functional expression of β**_**3**_**-AR showed mild polyuria and reduced urine osmolality.** (**a**) β_3_-adrenergic receptor–null mice (β_3_-AR^−/−^) and their age-matched controls (β_3_-AR^+/+^) (8 in each group) were individually housed in metabolic cages for 5 days, and 24-hour urine output, urine osmolality, and water intake were measured daily. The analysis reports the mean ± SEM values relative to 24-hour urine collection. In β_3_-AR^−/−^ mice, urine output was nearly 77% higher, urine osmolality 30% was lower, and water intake was 41% higher compared with control β_3_-AR^+/+^ mice. Statistical analysis was performed by unpaired *t*-test. *P < 0.05. (**b**) The same experimental protocol was applied to β_1-2_-AR-null mice (β_1-2_-AR^−/−^) and their age-matched controls (β_1-2_-AR^+/+^) (8 in each group). No statistically significant difference was observed in urine parameters and water intake between the 2 experimental groups. (**c**) Immunofluorescence analysis showed that β_3_-AR^−/−^ mice have reduced plasma membrane expression and higher subapical localization of AQP2 compared with control β_3_-AR^+/+^ mice (bar = 10 μm). (**d**) Immunofluorescence analysis with the antiphosphorylated Na-K-Cl cotransporter (pNKCC2) showed also that β_3_-AR^−/−^ mice have reduced levels of activated NKCC2 (pNKCC2) (bar = 30 μm). Comparable results were obtained in 3 different mice.

The effect of β_3_-AR agonism on AQP2 and NKCC2, the major players involved in antidiuresis, prompted us to investigate whether β_3_-AR inactivation may affect water and electrolyte handling in the kidney *in vivo*. To this end, we evaluated these parameters in β_3_-AR^−/−^ mice^29^ lacking β_3_-AR functional expression and β_1-2_-AR^−/−^ knockout mice,^30^ in which β_3_-AR is the only expressed β-AR. Age-matched wild-type (wt) mice of each strain were used as controls (β_3_-AR^+/+^ and β_1-2_-AR^+/+^). Strikingly, in β_3_-AR^−/−^, diuresis was higher (by 77%), urine osmolality was lower (by 30%), and water intake was increased (by 40%) compared with β_3_-AR^+/+^ (Figure 5a). In contrast, urine parameters and water intake were comparable between β_1-2_-AR^+/+^ and β_1-2_-AR^−/−^ mice (Figure 5b). No significant differences in food intake were observed between mouse strains (not shown).

In line with these results, immunofluorescence analysis showed that, compared with control β_3_-AR^+/+^ mice, β_3_-AR^−/−^ mice have reduced AQP2 plasma membrane expression and increased subapical localization (Figure 5c).

Analysis of urine electrolytes, reported in Table 1, showed that β_3_-AR^−/−^ mice have significantly higher urine excretion of Na^+^, K^+^, and Cl^−^ compared with their age-matched β_3_-AR^+/+^. Instead, the plasma concentration of the same electrolytes and the GFR were comparable between β_3_-AR^−/−^ and β_3_-AR^+/+^ mice. These results suggest reduced activity of the NKCC2 transporter in β_3_-AR^−/−^ mice.

Immunofluorescence analysis showed that in β_3_-AR^−/−^ mice, the antibody against phosphorylated NKCC2 detected a lower amount of activated NKCC2 in the outer medulla compared with β_3_-AR^+/+^ mice (Figure 5d).

**Figure S3:**
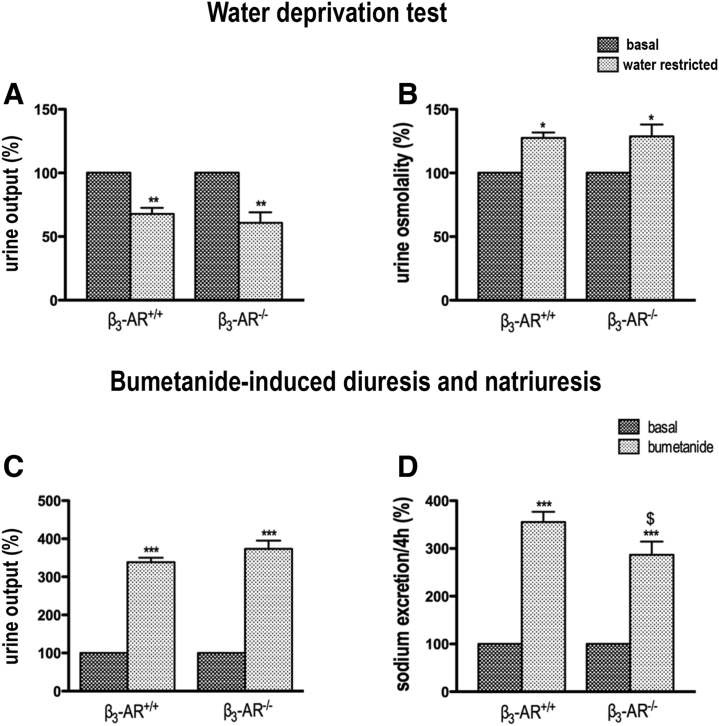
Water deprivation test and bumetanide-induced diuresis and natriuresis. β3-AR-null mice (β3-AR-/-) and their age-matched controls (β3-AR+/+) (N = 8 for each group), were individually housed in metabolic cages for 24 hours, then 4 animals per group were subjected to water deprivation for 24 hours, while 4 animals had free access to water (basal). The 24-hour urine output **(A)** and urine osmolality **(B)** of control animals were set as 100%. Urine output of water-deprived animals was reduced of 32% in β3-AR+/+ mice and of 40% in β3-AR-/- mice. Urine osmolality of water-deprived animals was increased of 27% in β3-AR+/+ mice and of 28% in β3-AR-/- mice. Data are provided as mean ± SEM. Significant differences between means were tested by one-way analysis of variance ANOVA with Newman-Keuls’s post-test. **P < 0.001, *P <0.05 No significant interstrain differences were observed. Urine output **(C)** and natriuresis **(D)** after the i.p. injection of vehicle or 40 mg/kg bumetanide for 4 hours in β3-AR+/+ and β3-AR-/- mice. The 4-hour urine output and urine Na+ excretion of control animals was set as 100% (n = 5). Data are provided as mean ± SEM. Significant differences between means were tested by one-way analysis of variance ANOVA with Newman-Keuls’s post-test. ***P < 0.0001 for intrastrain differences between vehicle and bumetanide treatments; §*P* < 0.01 for interstrain differences in the effects of bumetanide. The bumetanide-induced natriuresis was significantly attenuated in β3-AR-/- mice compared with β3-AR+/+.

To further support this evidence, we analyzed the effects of bumetanide injection on natriuresis in both β_3_-AR^−/−^ and β_3_-AR^+/+^ mice (Supplementary Figure 3c and d). Natriuresis was higher in β_3_-AR^+/+^ than in β_3_-AR^−/−^ mice (355.4 ± 21.55% vs. 287 ± 27.5%; *P* < 0.0001), confirming that β_3_-AR^−/−^ mice have less basal NKCC2 cotransporter activity to inhibit.

Of note, the maximal urine concentrating ability of β_3_-AR^−/−^ mice on a water deprivation test was comparable to that of β_3_-AR^+/+^ mice (Supplementary Figure S3a and b).

### Effect of β_3_-AR stimulation on urine output

**Figure 6:**
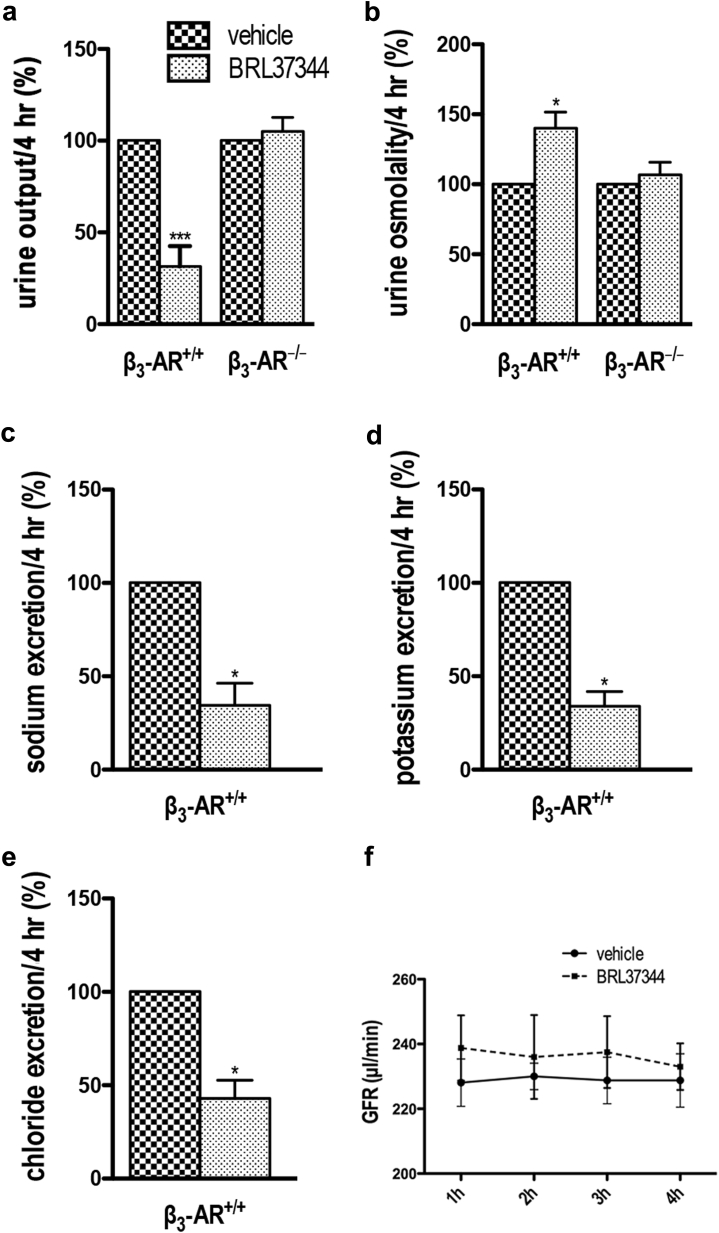
**Effect of β**_**3**_**-AR stimulation on urine concentrating ability in β**_**3**_**-AR**^**+/+**^**mice.** β_3_-adrenergic receptor (β_3_-AR)–null (β_3_-AR^−/−^) mice and their age-matched controls (β_3_-AR^+/+^) (10 of each genotype) were individually acclimatized in metabolic cages for 48 hours; 5 received a single i.p. injection of BRL37344 (BRL) (0.6 mg/kg), whereas 5 control animals received phosphate-buffered saline alone (vehicle). Urine samples were collected for 4 hours after injection. Urine output (**a**) and urine osmolality (**b**) were measured in β_3_-AR^+/+^ and β_3_-AR^−/−^ mice and expressed as a percentage of control values measured in vehicle-injected animals ± SEM. Urine output of β_3_-AR^+/+^ mice decreased ∼70% and urine osmolality increased ∼40% after BRL37344 (BRL) injection. No significant effect was seen in β_3_-AR^−/−^ mice. Significant differences between means were tested by 1-way analysis of variance with the Newman-Keuls posttest. *P < 0.05, ***P < 0.0001. (**c**,**d**,**e**) Urine excretion of Na^+^, K^+^, and Cl^–^, normalized for the urine volume, measured in β_3_-AR^+/+^ mice. Data are reported as a percentage of the values measured in vehicle-injected mice ± SEM. Significant differences between means were tested by the Mann-Whitney *U* test. *P < 0.05. (**f**) Glomerular filtration rate (GFR) of β_3_-AR^+/+^ conscious mice was measured at 1, 2, 3, and 4 hours after injection of BRL or vehicle alone. No significant difference was found at each time point between BRL- and vehicle-injected mice. Significant differences between means were tested by 2-way analysis of variance with a Bonferroni posttest.

Next, to uncover the possible antidiuretic effect of pharmacologic stimulation of β_3_-AR in mice, we examined whether BRL37344 could *per se* induce antidiuresis. β_3_-AR^+/+^ and β_3_-AR^−/−^ mice received a single i.p. injection of BRL37344 (0.6 mg/kg) or phosphate-buffered saline (PBS) alone (vehicle). Urine samples were collected for 4 hours after injections, the first time point at which all BRL37344-treated animals began to urinate. Diuresis, urine osmolality, and urine electrolyte excretion were analyzed and are shown in Figure 6. Notwithstanding the differing diuresis in β_3_-AR^−/−^ and β_3_-AR^+/+^ mice, we expressed our results as a percentage of the values measured in vehicle-treated animals of each genotype. Strikingly, BRL37344 greatly reduced the diuresis in β_3_-AR^+/+^ mice but not in β_3_-AR^−/−^ mice (Figure 6a). Concomitantly, BRL37344 significantly increased urine osmolality only in β_3_-AR^+/+^ mice (Figure 6b). Interestingly, in β_3_-AR^+/+^ mice, urine excretion of Na^+^, K^+^, and Cl^−^, normalized to the volume of diuresis, were significantly reduced by BRL37344 (Figure 6c–e). Of note, the GFR in β_3_-AR^+/+^ mice, measured at 1, 2, 3, and 4 hours after BRL37344 treatment, was not affected (Figure 6f).

### Effect of β_3_-AR stimulation on diuresis of mice lacking AVPR2

**Figure 7:**
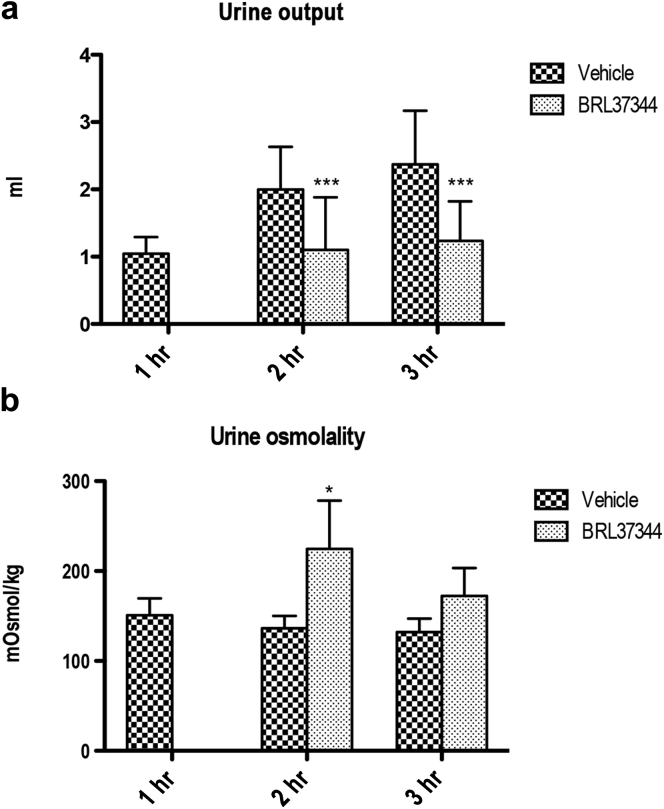
**β**_**3**_**-Adrenergic receptor stimulation promotes antidiuresis in mice lacking functional expression of the arginine vasopressin receptor type 2.** Ten *V2R*^*fl/y*^*Esr1-Cre* mice were acclimatized in mouse metabolic cages for 48 hours; 5 received a single i.p. injection of BRL37344 (0.6 mg/kg), whereas 5 control mice received phosphate-buffered saline alone (vehicle). Urine samples were collected every hour for 3 hours from both groups, and urine output (**a**) and urine osmolality (**b**) at each time point were reported. One hour after the injection, the urine output of BRL37344-treated mice was reduced to zero compared with vehicle-injected animals (vehicle) (diuresis, 1 hour). Two hours after injection, urine output of treated mice was still dramatically reduced compared with control animals (diuresis, 2 hours). Urine osmolality increased in BRL37344-injected animals (urine osmolality, 2 hours). At 3 hours postinjection, the effect of BRL on the urine output still persisted (urine output, 3 hours), whereas the effect on urine osmolality partially reversed (urine osmolality, 3 hours). The analysis reports the mean ± SEM values. Significant differences between measurements were tested by 2-way analysis of variance with a Bonferroni posttest for diuresis and by 1-way analysis of variance with a Bonferroni posttest. *P < 0.05; ***P < 0.001.

Next, we investigated whether the potent antidiuretic effect of BRL37344 observed in β_3_-AR^+/+^ mice could bypass the inactivation of the AVP signaling in mice lacking AVPR2.^12^, ^31^ Mice received a single i.p. injection of BRL37344 (0.6 mg/kg) or PBS alone (vehicle). Urine samples were collected every hour for 3 hours, and diuresis (Figure 7a) and urine osmolality (Figure 7b) were reported. Strikingly, 1 hour after the injection, the urine output of all BRL37344-treated mice was reduced to zero compared with vehicle-treated mice (Figure 7a, 1 hour). Therefore, we could not measure urine osmolality at this time point (Figure 7b, 1 hour). Two hours after injection, the diuresis of BRL37344-treated mice was still dramatically reduced compared with vehicle-treated animals (Figure 7a, 2 hours) and urine osmolality increased (Figure 7b, 2 hours). Three hours postinjection, the effect BRL37344 on diuresis still persisted (Figure 7a, 3 hours), whereas that on urine osmolality partially reversed (Figure 7b, 3 hours).

## Discussion

The possible expression and physiologic role of β_3_-AR in the kidney has not been investigated in depth thus far. The present results show β_3_-AR expression in the same AVPR2-expressing tubules. Because it is known that, similar to AVPR2, β_3_-AR activates the cAMP pathway,^32^ we hypothesized that pharmacologic stimulation of β_3_-AR might regulate the trafficking/activity of AQP2 and NKCC2 involved in the AVP-elicited antidiuresis in the kidney.^2^, ^3^ We first demonstrated that BRL37344 significantly increases cAMP production and promotes both AQP2 apical accumulation and NKCC2 phosphorylation/activation, suggesting that, similar to AVP, β_3_-AR agonists may increase reabsorption of water and solutes in the kidney.

The pharmacologic profile of BRL37344 indicates that it may have an intrinsic activity at β_1_-ARs or β_2_-ARs.^33^ As shown by the current results, BRL37344 effects on AQP2 and NKCC2 are prevented by the β_3_-AR antagonist L748,337 and are not observed in β_3_-AR^−/−^ mice, thus supporting the notion that BRL37344 acts selectively at β_3_-AR at the dose used and excluding an off-target effect.

Here we also show that β_3_-AR^−/−^ mice are characterized by mild polyuria, lower urine osmolality, and increased urinary excretion of Na^+^, K^+^, and Cl^−^ but not Ca^++^. Increased water excretion is in line with the observed reduced plasma membrane expression of AQP2 in the cortical collecting duct of β_3_-AR^−/−^. In addition, increased Na^+^, K^+^, and Cl^−^ excretion in β_3_-AR^−/−^ is in line with decreased NKCC2 activity, as also supported by the findings that β_3_-AR^−/−^ mice show less activated NKCC2 at the plasma membrane and a less pronounced natriuretic response to bumetanide.

The fact that food consumption in β_3_-AR^−/−^ mice is comparable to that of β_3_-AR^+/+^ mice (Table 1) seems to exclude that solute diuresis can explain the polyuria of β_3_-AR^−/−^ mice. Neither defect of AVP release (central polydipsia) can explain the polyuria of β_3_-AR^−/−^ mice because these mice show normal urine-concentrating abilities under a water deprivation challenge.

We also show that β_3_-AR^−/−^ mice have normal plasma levels of Na^+^, K^+^, Cl^−^, and Ca^++^ indicating that their polyuric phenotype is neither induced by hypercalciuria/hypercalcemia nor by hypokalemia.^34^, ^35^, ^36^ In addition, the polyuria in β_3_-AR^−/−^ mice is not a consequence of an increased GFR, which is comparable to that in β_3_-AR^+/+^ mice. On the other hand, β_1-2_-AR^−/−^ do not show alterations of urine output and osmolality, suggesting that β_3_-AR, rather β_1_-AR and β_2_-AR, regulate these urine parameters. However, the question whether β_3_-AR is more important than β_1_-AR and β_2_-AR in baseline renal function cannot be solved by the current study as we cannot compare the urine-concentrating ability of β_3_-AR^−/−^ and β_1-2_-AR^−/−^ mice. The 2 strains result from a different genetic background, and early studies showed that renal parameters significantly differ in mice of different strains.^37^

In line with the stimulatory effect of β_3_-AR activation on AQP2 subcellular localization and NKCC2 phosphorylation, BRL37344 exerts a potent antidiuretic effect in β_3_-AR^+/+^ mice but not in β_3_AR^−/−^ mice, thus confirming our *ex vivo* data on its specific action at the β_3_-AR. The additional finding that in β_3_-AR^+/+^ mice, BRL37344 reduces urinary excretion of Na^+^, K^+^, and Cl^−^ but not Ca^++^ and induces a 70% reduction of the urine output, whereas urine osmolality is increased by ∼40% may be explained by assuming that β_3_-AR stimulation promotes not only water but also salt reabsorption in the kidney. In line with this possibility, the strong reduction of urine output observed in BRL37344-treated mice is independent of the decrease in the GFR.

β_3_-ARs are also expressed in the hypothalamus^38^; thus, the possibility exists that the antidiuretic effect of BRL37344 may involve hypothalamic regulation of AVP release. Our results in AVPR2-null mice^31^ seem to exclude this possibility. In these mice, the classic symptoms of XNDI develop.^39^, ^40^, ^41^ As shown here, a single i.p. injection of BRL37344 greatly reduces the diuresis and increases urine osmolality, supporting the notion that, *in vivo*, β_3_-AR agonism triggers AVP-independent antidiuresis. In addition, results in live kidney slices, demonstrating that BRL37344 induces cAMP production, AQP2 plasma membrane accumulation, and NKCC2 phosphorylation/activation in the thick ascending limb of Henle, provide additional, although indirect, evidence that BRL37344 triggers its effect independently of central β_3_-AR activation.

The current results cannot exclude that the effects of BRL37344 on urine output may be related to the systemic effects of the drug on arterial pressure. However, it has been shown in rats that BRL37344 reduces arterial pressure by ∼14%^42^; therefore, it is unlikely that such an effect may be responsible for the observed 70% reduction in urine output.

In conclusion, our experimental data indicate that (i) in mice, β_3_-ARs are expressed in most of the AVP-sensitive nephron segments; (ii) β_3_-AR stimulation promotes AQP2 plasma membrane accumulation and NKCC2 activation, thus increasing water and salt reabsorption in the kidney tubule; (iii) this effect is likely mediated by an increase of intracellular cAMP; and (iv) β_3_-AR agonism induces antidiuresis in mice lacking AVPR2.

Taken together, these data suggest an unexplored role of sympathetic stimulation via the β_3_-AR in promoting antidiuresis under physiologic conditions. Some evidence indicates that there is a synaptic contact between renal sympathetic varicosities and renal tubular epithelial cell basolateral membranes.^12^, ^43^ In this respect, the current data support the hypothesis that sympathetic stimulation of β_3_-ARs, upregulating NKCC2 and AQP2 activity, can enhance solutes and water reabsorption in the nephron, thus eliciting an antidiuretic effect. Although we restricted our investigation to the regulatory role of β_3_-ARs on AQP2 and NKCC2, the possible effect of β_3_-AR stimulation on other Na/Cl transporters or additional AQPs, participating in the countercurrent multiplier system, is worth further investigation.

The observation that β_3_-AR^−/−^ mice are polyuric but show normal urine-concentrating ability during water deprivation suggest that, under physiologic conditions, β_3_-AR activation by sympathetic nerves does not provide an additional mechanism, corroborating the kidney antidiuretic response to AVP. Although much work remains to be done to fully understand the role of β_3_-ARs in water and salt reabsorption during sympathetic activation, the current results are potentially relevant for the development of novel pharmacologic approaches to the treatment of diseases caused by AVPR2-altered signaling, including XNDI, polycystic kidney diseases, and the syndrome of inappropriate secretion of AVP. For instance, in XNDI patients, β_3_-AR agonists may bypass the lack of AVPR2 function, restore NKCC2 and AQP2 activity, and improve the unpaired urine concentration mechanism. It must be emphasized that patients with autosomal forms of NDI^40^ due to mutations of the *AQP2* gene would not benefit from this potential treatment.

Further studies are needed to verify this proof-of-concept, but the ameliorative effect of BRL37344 on renal concentrating abilities of AVPR2-null mice strongly encourages studies in this direction. In particular, we suggest that agonists of the human β_3_-AR, such as mirabegron,^44^ already used to treat an overactive bladder, may either improve the impaired concentrating ability of the kidney or increase the beneficial effects of the current XNDI therapy.

## Materials and Methods

### Antibodies and reagents

Polyclonal antibodies against β_3_-AR (cat. nos. sc-50436 and sc-1473) were obtained from Santa Cruz Biotechnology (Dallas, TX) and were previously validated for Western blotting and immunofluorescence analysis.^45^, ^46^ Antibodies against AQP1 (cat. no. sc-20810), and CD-31 (cat. no. sc-1506), BRL37344 (cat. no. sc-200154), L748,337 (cat. no. sc-204044) were from Santa Cruz Biotechnology. H-89 (cat. no. B1427), and [deamino-Cys^1^, D-Arg^8^]-vasopressin (dDAVP, cat. no. V-1005) were from Sigma (St. Louis, MO). Antibody anti-CLC-K (cat. no. ACL-004) was from Alomone Labs (Jerusalem, Israel). Antibodies anti-NKCC2 (cat. no. AB3562P) were from Merck Millipore (Billerica, MA). Antibodies anti-NCC (cat. no. SPC-402D) were from StressMarq Biosciences Inc. (Victoria, BC, Canada). The antibody against human AQP2 was previously described.^47^ The antibody against the phosphorylated threonines 96 and 101 of phosphorylated mouse NKCC2^28^ was kindly provided by Prof. Biff Forbush, Yale University.

### β_3_-AR pharmacology

BRL37344 is a well-known β_3_-AR agonist^48^ that has been previously used in mice.^49^, ^50^, ^51^ BRL37344 displays a rank order of potency at the human β-ARs, that is, β_3_-AR > β_2_-AR > β_1_-AR, with an approximately 20-fold and 100-fold higher selectivity for β_3_-AR versus β_2_-AR and β_1_-AR, respectively.^52^ BRL37344 has been found to be effective at 10 μM in the human isolated internal anal sphincter model,^53^ in human retinal endothelial cells,^54^ and in mouse retinal explants.^55^

L748,337 has been reported as one of the very few antagonists with a high selectivity for β_3_-AR.^56^ Nonetheless, L748,337 remains the most suitable β_3_-AR antagonist currently available.^26^

### RNA isolation and reverse transcriptase polymerase chain reaction

Total RNA was extracted from mouse brown adipose tissue, the bladder, and the kidney by the TRIzol reagent and reverse-transcribed into cDNA using SuperScript VILO cDNA Synthesis Kit (Thermo Fisher Scientific, Waltham, MA).

The mouse β_3_-AR intron-spanning primers were previously reported.^20^ As a positive control, mouse β-actin cDNA was amplified using specific primers. Polymerase chain reaction was performed using Taq DNA polymerase recombinant (Life Technologies, Carlsbad, CA) according to the following: (94°C, 3 minutes) × 1 cycle and (94°C, 45 seconds; 55°C, 30 seconds; 72°C, 1 minute) × 40 cycles. Amplified products were analyzed on 3% agarose gel. Sequencing was performed by BMR Genomics (Padova, Italy), using the method of Sanger.

### Cell and tissue fractionation and immunoblotting

Brown adipose tissue, bladder, and kidney cortex/total medulla were isolated from male C57BL/6J mice and homogenized in radioimmunoprecipitation assay buffer.^57^ Where reported, kidney slices were lysed in antiphosphatase buffer.^2^

Fifteen micrograms of each lysate were separated by sodium dodecylsulfate-polyacrylamide gel electrophoresis and analyzed by Western blotting. After blocking with 3% bovine serum albumin, blots were incubated with anti–β_3_-AR antibody (sc-50436, 1:200) and anti–p-NKCC2 antibodies (1:1000) followed by horseradish peroxidase–conjugated secondary antibody.

Blots were revealed by enhanced chemiluminescence, with Chemidoc XRS, equipped with Image Lab Software (Bio-Rad, Hercules, CA) and quantified with ImageJ software.

### Immunofluorescence

Mouse kidneys were fixed with 4% paraformaldehyde in PBS at 4°C, dehydrated in graded ethanol, and embedded in paraffin wax. Serial sections, 5 μm thick, were deparaffinized, rehydrated, and subjected to immunofluorescence analysis. Antigen retrieval was performed by boiling sections in citrate buffer (10 mM sodium citrate, pH 6). After blocking with 1% bovine serum albumin in PBS for 30 minutes, sections were incubated with the primary antibodies β_3_-AR (sc-1473), AQP2, AQP1, CLC-K, NKCC2, CD31, NCC, and phosphorylated NKCC2.

Sections were incubated with AlexaFluor-conjugated secondary antibodies (Life Technologies). Confocal images were obtained with a confocal microscope (TSC-SP2, Leica; Wetzlar, Germany).

### Preparation of kidney tubule suspensions and cAMP assay

Kidneys from FVB/C57/129/DBA mice (10-week old males) were minced and enzymatically digested as previously reported.^10^ Aliquots of tubule suspensions were preincubated with the phosphodiesterase inhibitor IBMX for 10 minutes at 37°C. Subsequently, BRL37344 (1, 10, and 100 μM) or dDAVP (100 nM) were added, and reactions were carried out for 45 minutes at 37°C. Total intracellular cAMP was determined by enzyme-linked immunosorbent assay, as previously reported.^10^

### Kidney tissue slices: preparation and treatment

C57BL/6J male mice were anesthetized with tribromoethanol (250 mg/kg) and killed by cervical dislocation. Kidneys were excised, and thin transversal slices (250 μm) were cut using a McIlwain Tissue Chopper (Ted Pella Inc.; Redding, CA, United States). Slices were left at 37°C for 15 minutes in Dulbecco’s Modified Eagle Medium/F12 medium pre-equilibrated with 5% CO_2_, then stimulated for 40 minutes with dDAVP (10^−7^ M) or BRL37344 (10^−5^ M), the latter alone or after 30 minutes of preincubation with either L748,337 (10^−7^ M) or H89 (10^−5^ M). Slices were either processed for immunoblotting analysis or fixed in 4% paraformaldehyde and processed for immunofluorescence as described previously.

### Animal studies

All animal experiments were approved by the Institutional Committee on Research Animal Care, in accordance with the Italian Institute of Health Guide for the Care and Use of Laboratory Animals. Mice were maintained on a 12-hour light/12-hour dark cycle, with free access to water and food.

β_3_-AR^−/−^ and β_3_-AR^+/+^ mice^58^ were purchased from Jackson Laboratory (Bar Harbor, ME, United States). β_1-2_-AR^−/−^ and β_1-2_-AR^+/+^ mice^30^ were generated as previously described.^59^, ^60^ Metabolic cages were used to measure urine output, osmolality, and water intake. Mice received a single i.p. injection of BRL37344 (0.6 mg/kg) or PBS alone. Electrolytes were measured using the ion selective electrode method.

The GFR of conscious mice was measured as previously reported.^61^ AVPR2 knockout mice (V2R^*fl/fl*^ and V2R^*fl/y*^ Esr1-Cre mice) were previously described.^31^

V2R^*fl/y*^ Esr1-Cre mice received a single i.p. injection of BRL37344 (0.6 mg/kg) or PBS alone and urine output and osmolality were monitored every hour for 3 hours. Urine osmolality was measured using a vapor pressure osmometer.

### Statistical analysis

For statistical analysis, GraphPad Prism software (La Jolla, CA) was used. The statistical analysis performed is indicated in the figure legends.

## Disclosure

All the authors declared no competing interests.

## Figures and Tables

**Table 1:** Plasma electrolyte concentrations, renal 24-h electrolyte excretion, GFRs, and food intake in β_3_-AR^+/+^ and β_3_-AR^−/−^ mice

	Electrolytes	β_3_-AR^+/+^	β_3_-AR^−/−^	*P* Value
Plasma				
	Na^+^ (mEq/l)	139.0 ± 5.57	141.3 ± 0.67	NS
	K^+^ (mEq/l)	6.73 ± 0.29	6.43 ± 0.19	NS
	Cl^**−**^ (mEq/l)	108.0 ± 3.22	106.3 ± 2.67	NS
	Ca^2+^ (mEq/l)	3.16 ± 0.53	3.34 ± 0.44	NS
Urine				
	Na^+^ (mEq/24 hr)	019 ± 0.02	0.27 ± 0.01	*P* < 0.01
	K^+^ (mEq/24 hr)	0.18 ± 0.02	0.23 ± 0.01	*P* < 0.05
	Cl^**−**^ (mEq/24 hr)	0.45 ± 0.04	0.59 ± 0.03	*P* < 0.01
	Ca^2+^ (mEq/24 hr)	0.005 ± 0.0006	0.005 ± 0.0005	NS
	GFR (μl/min)	235.5 ± 20.76	253.8 ± 30.94	NS
	Food intake (g)	5.07 ± 0.08	5.14 ± 0.08	NS

Values are means ± SEM of measurements in 8 mice/genotype. Statistical analysis was performed using an unpaired *t* test.

GFR, glomerular filtration rate; NS, not significant.
